# A Simple and Effective Mass Spectrometric Approach to Identify the Adulteration of the Mediterranean Diet Component Extra-Virgin Olive Oil with Corn Oil

**DOI:** 10.3390/ijms160920896

**Published:** 2015-09-01

**Authors:** Francesco Di Girolamo, Andrea Masotti, Isabella Lante, Margherita Scapaticci, Cosima Damiana Calvano, Carlo Zambonin, Maurizio Muraca, Lorenza Putignani

**Affiliations:** 1 Department of Laboratory Medicine, Bambino Gesù Children’s Hospital, Scientific Institute for Research, Hospitalization and Health Care (IRCCS), Piazza Sant’Onofrio 4, Rome 00165, Italy; 2 Gene Expression-Microarrays Laboratory, Bambino Gesù Children’s Hospital, Scientific Institute for Research, Hospitalization and Health Care (IRCCS), Piazza Sant’Onofrio 4, Rome 00165, Italy; E-Mail: andrea.masotti@opbg.net; 3 Department of Laboratory Medicine, San Camillo Hospital, Viale Vittorio Veneto 18, Treviso 31100, Italy; E-Mails: Isa.Lante@gmail.com (I.L.); scapaticci.m@gmail.com (M.S.); 4 Dipartimento di Chimica, Università degli Studi di Bari “Aldo Moro”, Via Orabona 4, Bari 70126, Italy; E-Mails: cosimadamiana.calvano@uniba.it (C.D.C.); carlo.zambonin@uniba.it (C.Z.); 5 Department of Women’s and Children’s Health, University of Padova, Via Giustiniani 3, Padova 35122, Italy; E-Mail: muraca@unipd.it; 6 Parasitology Unit, Bambino Gesù Children’s Hospital, Scientific Institute for Research, Hospitalization and Health Care (IRCCS), Piazza Sant’Onofrio 4, Rome 00165, Italy; 7 Metagenomics Unit, Bambino Gesù Children’s Hospital, Scientific Institute for Research, Hospitalization and Health Care (IRCCS), Piazza Sant’Onofrio 4, Rome 00165, Italy

**Keywords:** mediterranean diet, extra-virgin olive oil, corn oil, MALDI-TOF MS, phospholipids’ profiles

## Abstract

Extra virgin olive oil (EVOO) with its nutraceutical characteristics substantially contributes as a major nutrient to the health benefit of the Mediterranean diet. Unfortunately, the adulteration of EVOO with less expensive oils (e.g., peanut and corn oils), has become one of the biggest source of agricultural fraud in the European Union, with important health implications for consumers, mainly due to the introduction of seed oil-derived allergens causing, especially in children, severe food allergy phenomena. In this regard, revealing adulterations of EVOO is of fundamental importance for health care and prevention reasons, especially in children. To this aim, effective analytical methods to assess EVOO purity are necessary. Here, we propose a simple, rapid, robust and very sensitive method for non-specialized mass spectrometric laboratory, based on the matrix-assisted laser desorption/ionization mass spectrometry (MALDI-TOF MS) coupled to unsupervised hierarchical clustering (UHC), principal component (PCA) and Pearson’s correlation analyses, to reveal corn oil (CO) adulterations in EVOO at very low levels (down to 0.5%).

## 1. Introduction

Extra virgin olive oil (EVOO) is a fat made by crushing olives and extracting the juice from the *Olea europaea* (*i.e*., *Oleaceae* family) a traditional tree crop of the Mediterranean region.

Remarkably, EVOO represents the principal source of fat in the countries of the Mediterranean basin [1], historically associated with good health and longevity [2]. EVOO also contains an important family of bioactive compounds represented by carotenoids, sterols, lycopene, and hydrophilic phenols (oleuropein, oleocanthal, hydroxytyrosol, and tyrosol) [3].

Moreover, EVOO has anti-oxidant activity due to the presence of polyphenols and vitamins A–E and K, and may reduce risk factors of coronary heart disease, prevent several varieties of cancers and modify immune and inflammatory responses [4,5].

EVOO contains high levels of monounsaturated fatty acids (MUFAs) (considered a healthy dietary fat) and a higher MUFA/saturated FAs (SFAs) ratio. In the long term, these properties contribute to the protective effects by lowering “bad” cholesterol and raising “good” cholesterol [6,7]. Indeed, the consumption of olive oil, as the predominant fat intake, provides high oleic acid content and polyphenols, which have atherogenic, antioxidant and anti-inflammatory effects and reduce the cholesterol/high density lipoprotein (HDL) ratio and the concentration of the oxidized low-density lipoprotein (LDL) [8,9,10].

Several studies demonstrated that diets with MUFA-rich EVOO can reduce the risk of obesity in childhood [11,12]. Moreover, due to the high level of FAs and fat-soluble vitamins, EVOO is a source of high-density energy and is sometimes recommended in case of premature birth, a condition that requires a large amount of calories in a small quantity of food [13].

The Mediterranean diet (MD) principal protective compounds against diabetes are contained in fibers and vegetable fats; in particular, this protection is guaranteed by EVOO intake (rich in MUFAs) used for cooking, spreading, dressing and frying foodstuffs [14,15].

The presence of oleic, linoleic, and linolenic acids contribute to the development and growth of baby’s bones and brains [16]. Indeed, EVOO and some other components of the MD) (*i.e.*, walnuts and moderate quantities of wine), or foods with antioxidant properties or rich in polyphenols, are independently associated with better cognitive function and high plasma levels of ω-3 FAs [17]. This evidence suggests that the association between MD adherence and cognitive functions may be mediated by vascular factors, but also by non-vascular biological mechanisms, such as oxidative stress, inflammation and metabolic disorders [18], supporting the importance of MD in health [15,19], aging and lifestyle [20].

Moreover, EVOO and other vegetable fats, containing polyunsaturated FAs (PUFAs), are reported as inversely correlated to upper digestive, stomach and urinary tract cancer development risk [21].

According to European Union (EU) Regulation EC 1531/2001, the labelling of “extra-virgin” is allowed only if oil is cold-pressed and does not contain any trace of refined oil or other oleaginous seeds oil. Therefore, EVOO is the only cooking oil that is made without the use of chemicals and industrial refining. To this purpose, EVOO preserves a fine aroma and a pleasant taste and is worldwide appreciated for its nutritional value and health benefits [4,22,23] being considered as a nutraceutical [24].

The adulteration of EVOO with less expensive seed oils to increase yield and commercial profits is frequent [25] and the cross contamination of EVOO with other cooking seed oils during production that introduce oil-derived allergens may sometimes have severe downstream health implications for consumers in their diet, especially in children, potentially causing food allergies [26].

CO is a seed oil used for cooking. CO contains polyunsaturated fatty acid, vitamin E and also allergenic proteins that may elicit an immunoglobulin E (IgE)-mediated corn allergy [26].

In this regard, revealing adulterations of EVOO is of fundamental importance for health reasons. Therefore, innovative, simple and low cost analytical procedures to assess EVOO purity are desirable.

Currently, several analytical methods have been used in order to detect such adulteration/contamination principally based on chromatographic analyses [27,28], differential scanning calorimetry [29] nuclear magnetic resonance [30,31,32] and mass spectrometry [33,34,35,36].

Numerous studies have been devoted to the analysis of oil compounds such as sterols [37], triacylglycerols [38] or phospholipid [34]. The latter approach has demonstrated to be promising since the concentration range of phospholipids (PLs) (10–20 g/kg) in seed oils [39], is usually 300–400 times higher than in olive oils [40].

On the basis of the above considerations, here we present a simple, rapid, robust and very sensitive method for non-specialized mass spectrometric laboratory. Our approach exploits the performance of linear MALDI-TOF MS, coupled to unsupervised hierarchical clustering (UHC), principal component (PCA) and Pearson’s correlation analyses, for the detection of CO adulteration in EVOO at very low levels (down to 0.5%), with the final aim of preserving the intrinsic nutrition value of such a fundamental MD food.

To this aim, pure oil samples or binary mixtures containing EVOO with CO, were prepared according to Calvano *et al.* [41], for selective polar lipid enrichment and direct analyses by MALDI-TOF MS.

## 2. Results and Discussion

### 2.1. Analytical Performance of MALDI-TOF MS Lipid Profiles in Discriminating Corn Oil

**Figure 1:**
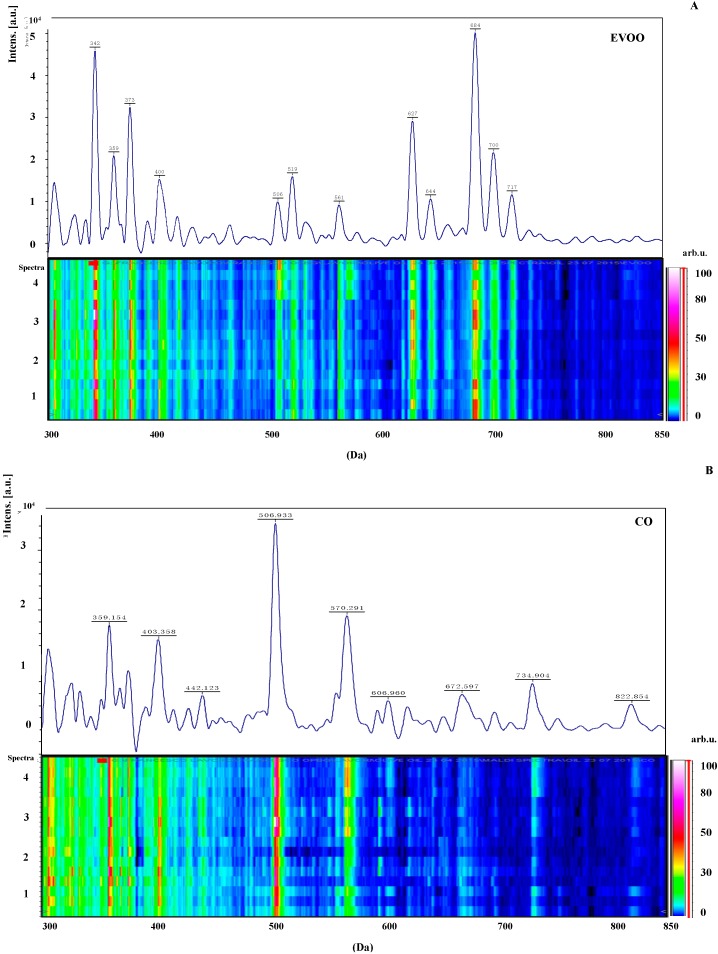
Pseudo-gel like and MALDI-TOF MS lipid profiles of EVOO (**A**) and CO (**B**). The mass values (*m*/*z*) are reported on the *X*-axis, while the color bar on the *Y*-axis indicates the peak intensity. Discriminating mass peaks have *m*/*z* values below 850.

Four independent MALDI-TOF MS lipid profiles (technical replicates) from each EVOO (four different EVOO) and CO (four different CO) samples, were visually inspected and the resulting flattened spectra profiles compared by gel-like representations (Figure 1). Raw data of all of these spectra have been extracted for further statistical elaboration. It is worth noting that when using a conventional matrix such as 2,5-dihydroxybenzoic acid (DHB), no significant differences were noticed among EVOO and CO, suggesting that only minor lipid components are likely to be found enriched for discriminating among oils.

In this regard, recently, Calvano *et al.* [41] reported that by using the ionic liquid tributylamine (TBA)-α-Cyano-4-hydroxycinnamic acid (CHCA) the signals relevant to phospholipids increased, allowing the discrimination of hazelnut oil from olive oil samples. Also in the herein presented linear MALDI-TOF MS spectra, the lipid profiles of CO seed oils were different from EVOO samples, the latter lacking signals in the 700–850 *m*/*z* mass range.

According to a previous study, virgin olive oils contain phospholipids in the range of 40–135 mg/kg [12]; this is in contrast to seed oils, for which the phospholipid content was 1000 times higher than EVOO [42]. Phosphatidylcholine, phosphatidylethanolamine, phosphatidylinositol and phosphatidylserine were reported to be the main phospholipids present in olive oil using thin-layer chromatography [13]. In a recent study [43], phosphatidylglycerol was the major phospholipid found in EVOO by liquid chromatography-mass spectrometry (LC–MS) as observed also by MALDI-TOF MS [41]. The presence of phosphatidylcholine and phosphatidylinositol was reported in corn oil [44].

**Figure 2:**
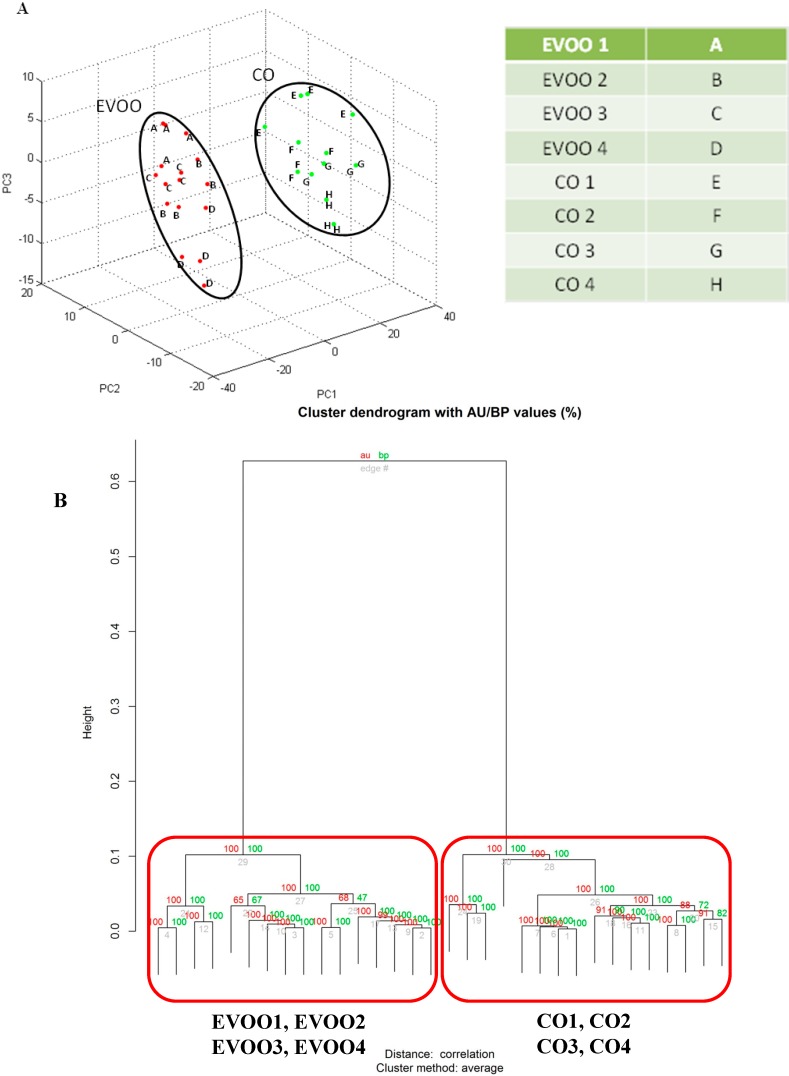

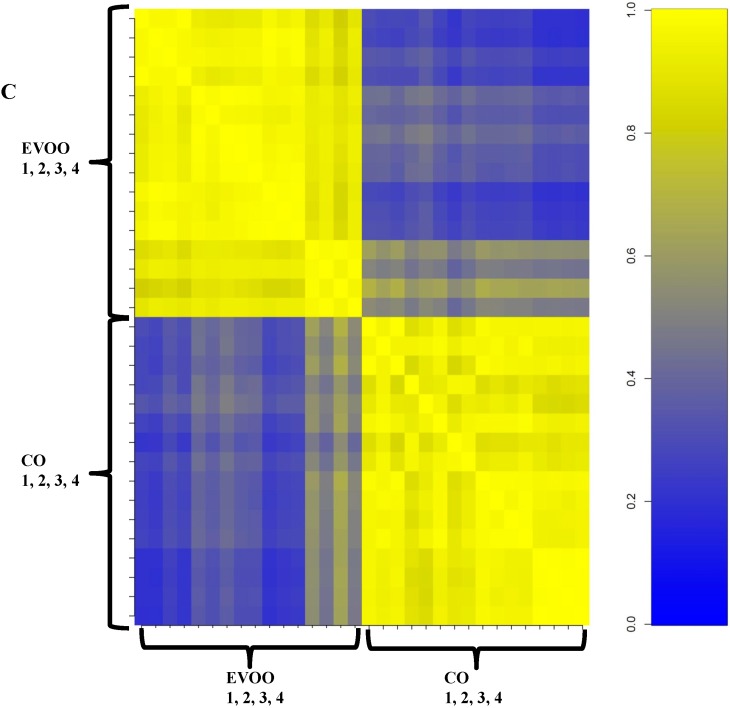
Data analysis of independent MALDI-TOF MS lipid spectra profiles of EVOO and CO. (**A**) Shows the 3D scatter plot image from the PCA analysis obtained by Biotyper software for EVOO and CO samples; (**B**) Shows the UHC tree (bootstrap *n* = 1000) generated by pvclust of each EVOO, and CO sample, and their replicates. Values on the edges of the clustering are *p*-values (%). Red values are AU (Approximately Unbiased) *p*-values, and green values are BP (Bootstrap Probability) values, as explained in experimental section; (**C**) Shows Pearson’s correlation coefficients represented as a correlation matrix of e EVOO and CO samples and their replicates. The correlation coefficients have been colored according to a scale ranging from 0 to 1, where blue corresponds to 0 and yellow to 1.

Subsequently, spectra were analysed by unsupervised hierarchical clustering (UHC) and principal component analysis (PCA) (Figure 2A) using the integrated software Biotyper 3.1 (Bruker Daltonics, Bremen, Germany). This software allows only a simple classification analysis and does not allow the optimization of statistical parameters or calculation of the reproducibility of this technique. Therefore, spectra profiles were clustered and bootstrapped (*n* = 1000) (Figure 2B) and correlation matrices (Figure 2C) were calculated with external statistical software (R Bioconductor).

According to the PCA representation (Figure 2A) and the bootstrapped (*n* = 1000) hierarchical clustering tree (Figure 2B) generated from the replicated MS profiles of the four oils, we found that all the mass spectra replicates for the two oils closely clustered together, whereas EVOO and CO results were well separated from each other, CO clustered with EVOO in a second clade (D = 0.63).

Finally, the Pearson’s correlation matrix (Figure 2C) and the correspondent correlation values reported in the Table S1, confirmed the good intra-species correlation (at the level of the technical replicates of the same oil sample and between the different brands) and the net inter-species separation as previously shown in Figure 2A,B. Conversely, a low inter-group correlation especially between EVOO and CO was observed.

These data outline the high reproducibility and effectiveness of MALDI-TOF MS analysis combined to specific data analysis tools for the characterization of EVOO and CO oils and their mixtures.

### 2.2. Analytical Performance of Polar Lipid MALDI-TOF MS Profiles in Discriminating the EVOO Adulteration with CO

In order to assess the performance of the proposed analytical workflow to identify EVOO adulteration at a low level of contamination, five mixtures were prepared by adding CO to EVOO at different percentages (0.5%, 1%, 5%, 10%, 20% *w*/*w*). Five replicas for each oil mixture were analysed. The MALDI-TOF mass spectra profiles were acquired in the 300–1300 *m*/*z* mass range. EVOO adulteration with CO was analyzed by Pearson’s correlation analysis (Table 1).

**Figure 3:**
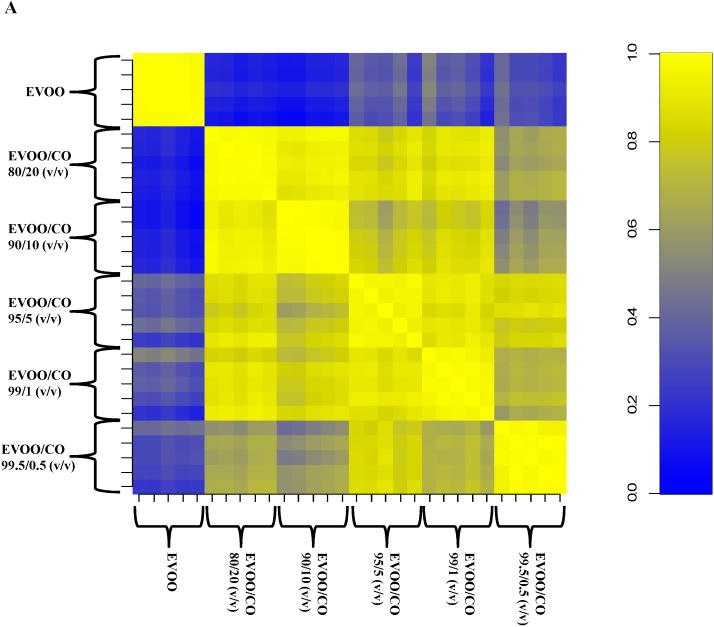

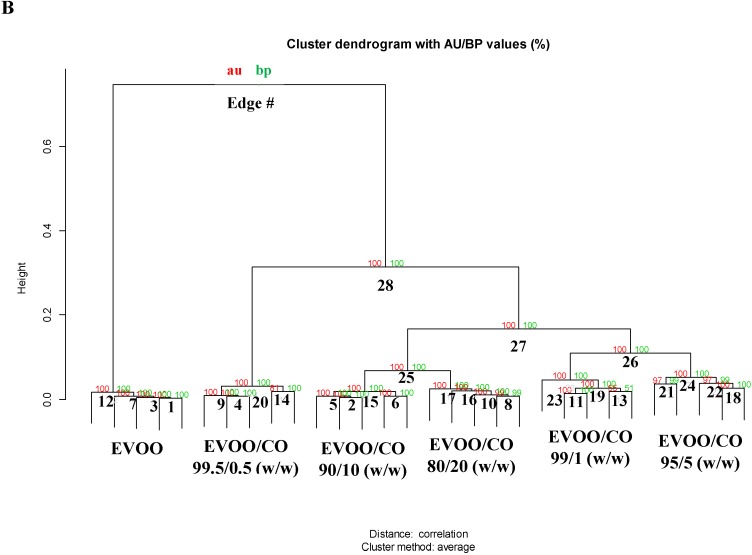
Pearson’s correlation matrix (**A**) and UHC tree (bootstrap *n* = 1000) (**B**) generated via pvclust of all spectral replica for the five mixtures (from 20% to 0.5%) of simulated adulterations of EVOO with CO. Correlation coefficients are represented with decreasing blue and yellow colors according to a scale ranging from 0 to 1, respectively. Values on the edges of the clustering are *p*-values (%). Red values are AU (Approximately Unbiased) *p*-values, and green values are BP (Bootstrap Probability) values as explained in the Experimental section.

The Pearson’s correlation matrix representation for EVOO adulteration with CO (Figure 3A) showed a high intra-class similarity (replica in the diagonal) and a variable inter-class correlation (Table 1). In particular, the mean correlation coefficients spanned from a minimum of 0.13–0.14 (for 10%–20% adulteration) up to 0.31–0.35 (for 0.5%–5% adulteration), in agreement with the expected behavior of mixtures containing a decreasing amount of adulterant. Clustering analysis showed a good separation of the different groups of spectra, corresponding to the different percentages of adulteration.

**Figure 4:**
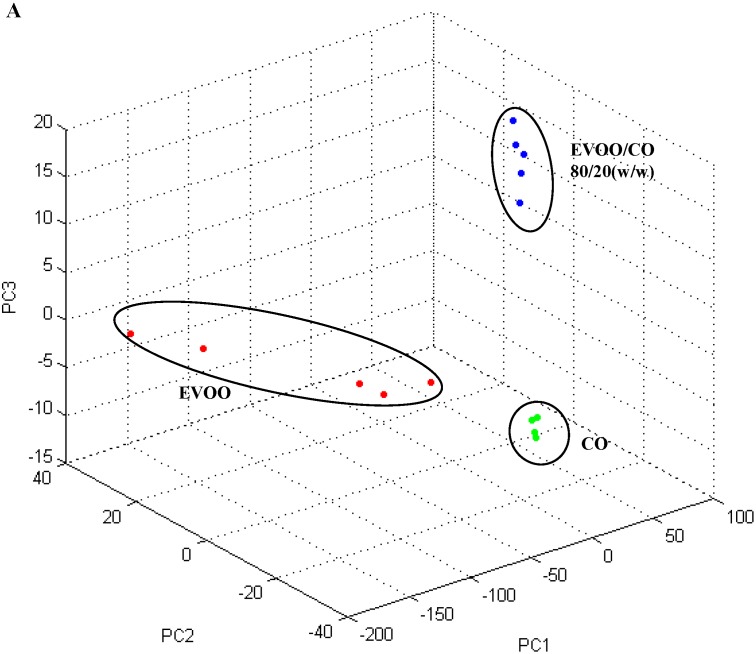

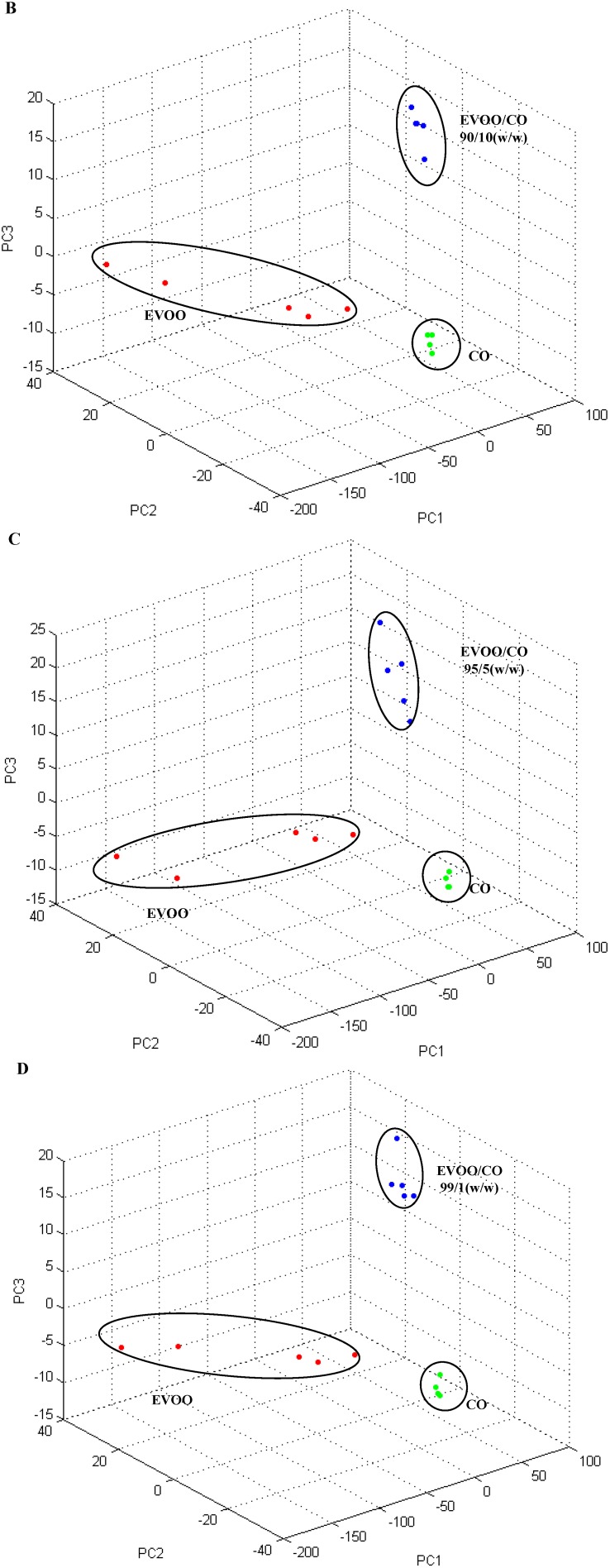

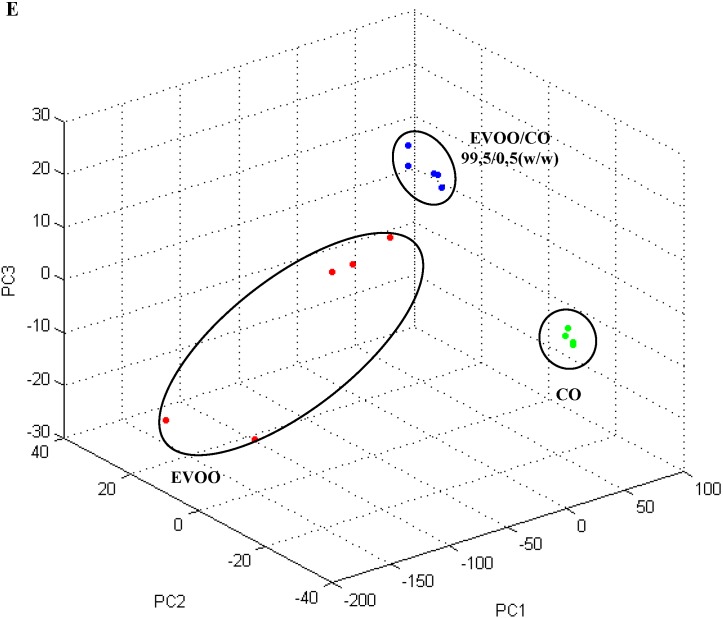
3D scatter plots of the first three PC for the five mixtures (20%, 10%, 5%, 1% and 0.5%), obtained by adding CO (**A**–**E**) to EVOO. All EVOO, CO and oil adulterations replicas grouped inside correct cluster.

Subsequently, we grouped all spectra, from EVOO adulteration/contamination, within consistent PCA clusters for EVOO/CO (Figure 4, Panels A–E) mixtures.

Hierarchical clustering is a 2D representation of the Euclidean distance between the various spectra, whereas PCA is a 3D representation where suitable principal components can be properly selected to group similar samples. The advantage of PCA is the possibility to visually rotate the PC graph for a better inspection of the acquired samples. The PCA analysis obtained by ClinProTools™ software confirmed the good separation between spectra associated to the groups of different percentages of CO adulteration.

**Table 1:** Mean Pearson’s correlation coefficients of different adulteration percentages (CO adulteration of EVOO).

Adulteration	20/80 (*v*/*v*)	10/90 (*v*/*v*)	5/95 (*v*/*v*)	1/99 (*v*/*v*)	0.5/99.5 (*v*/*v*)
CO/EVOO	0.139	0.127	0.346	0.340	0.311

To verify the robustness of our analytical method, we prepared other two blinded CO/EVOO mixtures (volume ratios, 20/80, 0.5/99.5) and another investigator analyzed them (independent blind analysis) and identified both the adulteration levels by means of ClinProTools software (Figure S1).

## 3. Experimental Section

### 3.1. Synthesis of the Ionic Liquid Matrix Tributylamine-α-cyano-4-hydroxycinnamic Acid (TBA-CHCA)

The ionic liquid matrix was prepared accordingly to Armstrong *et al.* procedure [45]. Briefly, organic salt of CHCA was prepared by dissolving 0.25 g of CHCA in 7.5 mL of methanol (MeOH); an equimolar amount of tributylamine was added and the mixture was sonicated (5 min) and, thus, filtered. The solvent was evaporated and the TBA-CHCA was dried at room temperature (RT) under vacuum overnight until the last traces of the solvent were removed. Then, the viscous ionic liquid of TBA-CHCA was dissolved in MeOH at 1.2 mM concentration and used for the subsequent experiments.

### 3.2. Oil Sampling and Pre-Treatment

Four pure EVOOs (homemade by four different Italian cultivars, Lazio and Puglia) and commercial pure COs (four different brands) were analysed. Samples were stored in dark bottles without headspace at RT before analyses. EVOO was intentionally adulterated by CO at different percentages (CO/EVOO mixtures in volume ratios 0.5/99.5, 1/99, 5/95, 10/90, 20/80).

As described by Calvano *et al.* [46], oil samples (0.5 mL) were treated with 1.87 mL of CHCl_3_/TBA-CHCA (1:2) and followed by vigorous vortex-mixing. Then, 0.62 mL of CHCl_3_ and 0.62 mL of water were added and vortexed after at each addition. The solution was centrifuged (15 min at 2000× *g*) and 1 µL of the organic layer was directly spotted onto the target plate and analyzed by a Microflex LT linear mass spectrometer (Bruker Daltonics, Bremen, Germany), for the generation of lipidomic phenotyping oil profiles as reported in the next paragraph. Each oil and adulterated oil sample has been treated five times to evaluate the method reproducibility and each sample has been acquired five times to evaluate the precision of the mass spectral measurements.

### 3.3. MALDI-TOF MS Spectra Acquisition

Spectra were recorded in the positive linear mode (laser frequency, 20 Hz; ion source 1 voltage, 20 kV; ion source 2 voltage, 18.4 kV; lens voltage, 9.1 kV; mass range, 300 to 1300 Da), using the FlexControl software package (version 3.0 Bruker Daltonics) [46]. Five independent spectra (500 shots one step from different positions of the target spot for spectrum) from each oil sample were manually collected, externally calibrated by using Bacterial Test Standard (Bruker Daltonics) and subsequently analyzed.

### 3.4. MALDI-TOF MS Spectra Analysis

In our experimental setup, up to 67 mass spectra were collected (4 replicates spectra for four oil species of two different sources, EVOO and CO, and five replicates spectra for five different percentage of adulteration for CO).

MS spectra were manually acquired, visually inspected before statistical analysis and loaded into FlexAnalysis software, version 3.0 (Bruker Daltonics) to perform the following pre-process analysis: (i) mass adjustment, spectra were compressed by a factor of 10 in the total mass range; (ii) smoothing, mass data were adjusted by the Savitsky-Golay algorithm with a frame size of 25 Da; (iii) baseline subtraction, was applied the minimum value for finding the baseline; (iv) normalization, was applied the maximum norm to normalize the baseline subtracted data; (v) peak picking, was applied spectra differentiation algorithm for finding the peaks, maximum peaks 100, threshold 0.1, method Peak Fitting.

Subsequently, the total pre-processed raw datasets of the 32 spectra replica from EVOO, CO oil samples and 35 spectra replica from the intentional adulterated EVOO with CO were imported in R Bioconductor (http://www.bioconductor.org/) [47] for Pearson’s correlation analysis and UHC. The package pvclust was applied for bootstrapping. For each cluster in UHC, *p*-values (between 0 and 1) were calculated via multiscale bootstrap resampling. Two different *p*-values were provided by the package pvclust: AU (Approximately Unbiased) and BP (Bootstrap Probability). AU is computed by multiscale bootstrap resampling and is a better approximation to unbiased *p*-value than BP value computed by normal bootstrap resampling. The same pre-processed raw datasets were imported into ClinProTools™ bioinformatics software (version 2.2 Bruker Daltonics, Bremen, Germany) [48] and converted into a virtual gel-like format. The mass values (*m*/*z*) were reported on the *X*-axis, while the color bar, reported on the *Y*-axis, showed the relationship between the color and the peak intensity.

Finally, PCA was performed via ClinProTools™ software that employs for the calculation only those peaks with statistically significant results after group classification. On the basis of a Welch’s *t*-test, a *p* value for each peak was calculated. This value indicates the probability that the observed intensity differences among the various peaks are due to chance. These calculations have been done independently for peak heights and peak areas. A list of all peaks sorted according to the statistical significance have been reported in the Table S2 and the discriminating peak of the adulteration mixtures is represented in the Figure S2.

## 4. Conclusions

Many people worldwide (including children) usually consume EVOO, a fundamental food in the MD that has the potential to prevent serious illnesses such as allergies, heart diseases, cancer and respiratory diseases.

Our pediatric hospital has established many diet-based prevention programs to monitor children’s health and to suggest correct eating and lifestyle behaviours, and an example is represented by a recent national initiative (http://www.nutrirelavita.it/). In this context, MALDI-TOF methods for the identification of EVOO adulterations can be recognized not only as a useful “foodomics” tool but also as an effective technique to control the quality of food components and, ultimately, to prevent food allergies.

We demonstrated that a simple and effective mass spectrometry method based on MALDI-TOF MS coupled to UHC and PCA analyses is able to characterize/type oils and allow an accurate and sensitive investigation of the most common adulterants. This approach does not involve laborious pre-analytical sample separation steps and may represent a fast, reliable and robust method for routine analyses of EVOO adulterations down to 0.5% limits for a non-specialized mass spectrometric laboratories.

## Supplementary Materials

Supplementary materials can be found at http://www.mdpi.com/1422-0067/16/09/20896/s1.
