# Supplementary material for: A Simple and Effective Mass Spectrometric Approach to Identify the Adulteration of the Mediterranean Diet Component Extra-Virgin Olive Oil with Corn Oil
Source: Int J Mol Sci. 2015 Sep 1;16(9):20896–912. doi: 10.3390/ijms160920896 (PMC4613234; doi:10.3390/ijms160920896)
Supplement: Supplementary file 1 [file ijms-16-20896-s001.zip › ijms-87047-Supplementary Information/ijms-87047-Supplementary Figure S1 and S2.pdf]

Supplementary Information

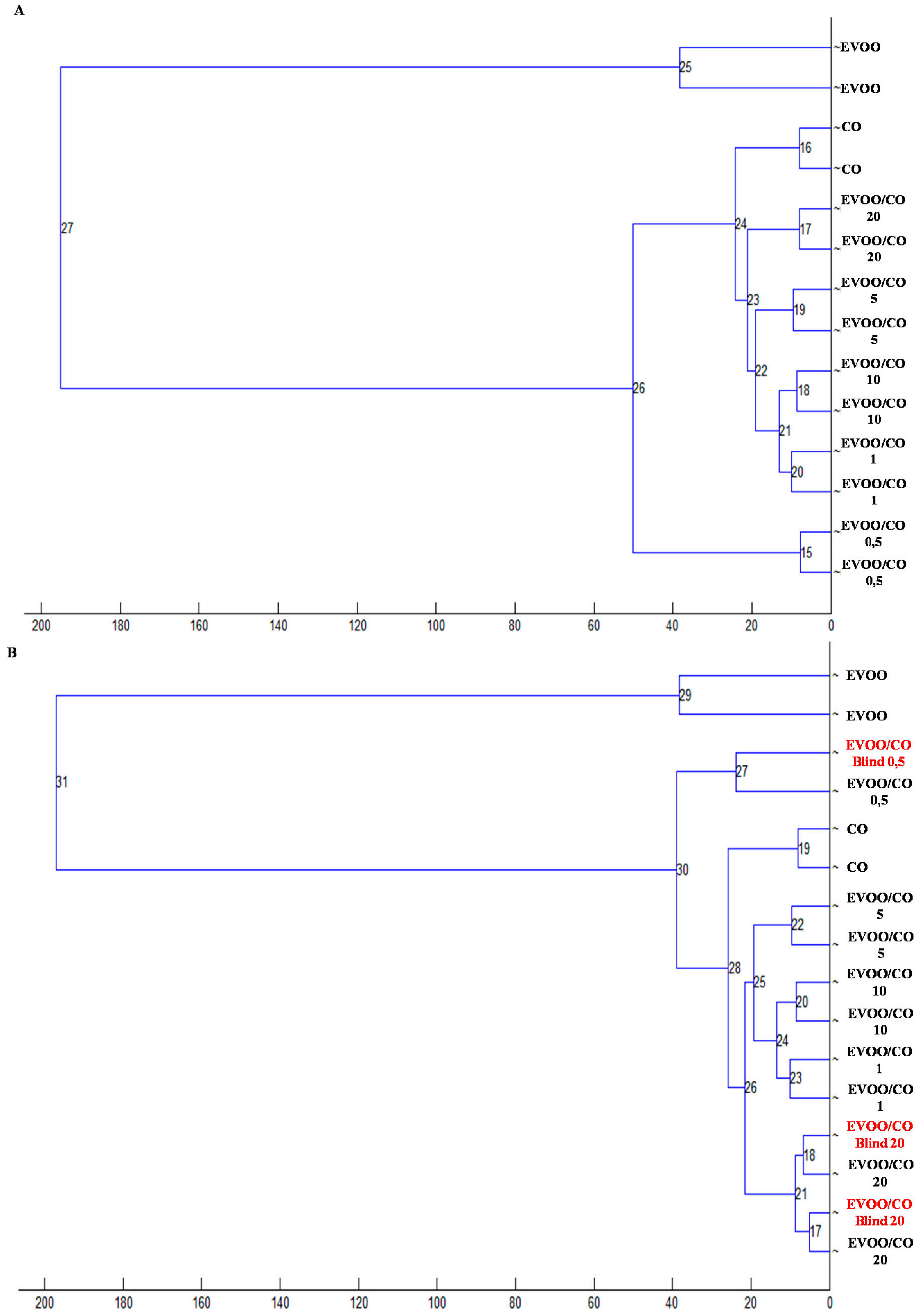

Figure S1. Two blinded CO/EVOO mixtures (**A**,**B**) were prepared (volume ratios, 20/80, 0.5/99.5) and another investigator analyzed them (independent blind analysis) and identified both the adulteration levels (red) by means of ClinProTools software.

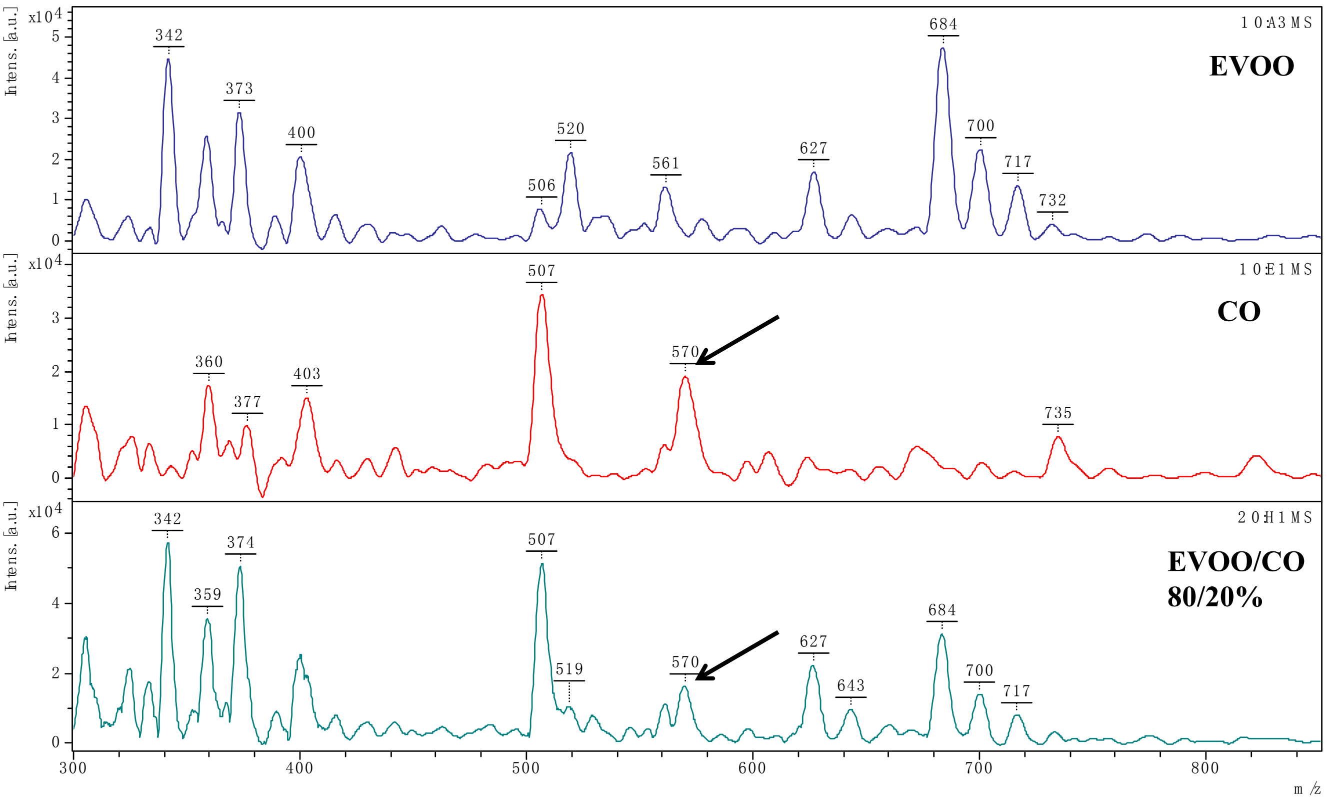

(A)

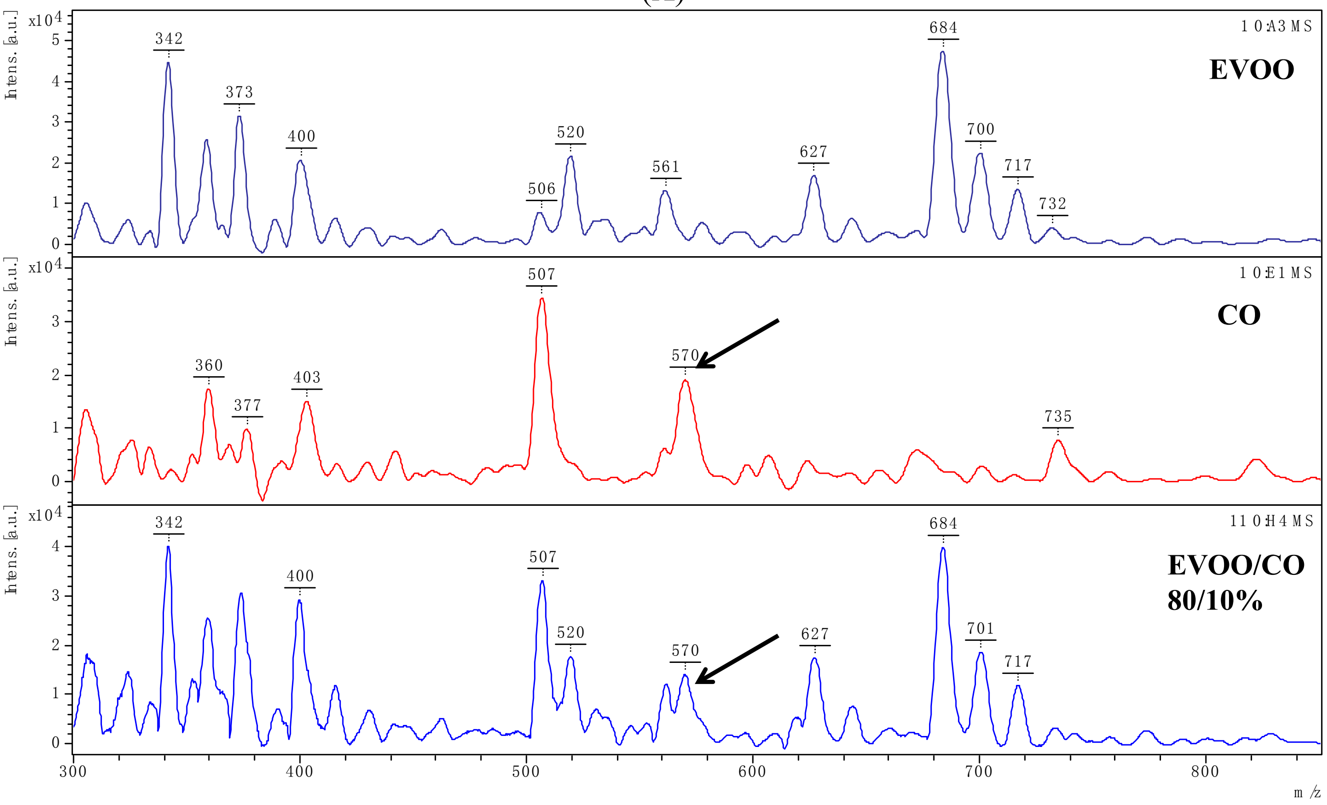

(B)

Figure S2. *Cont.*

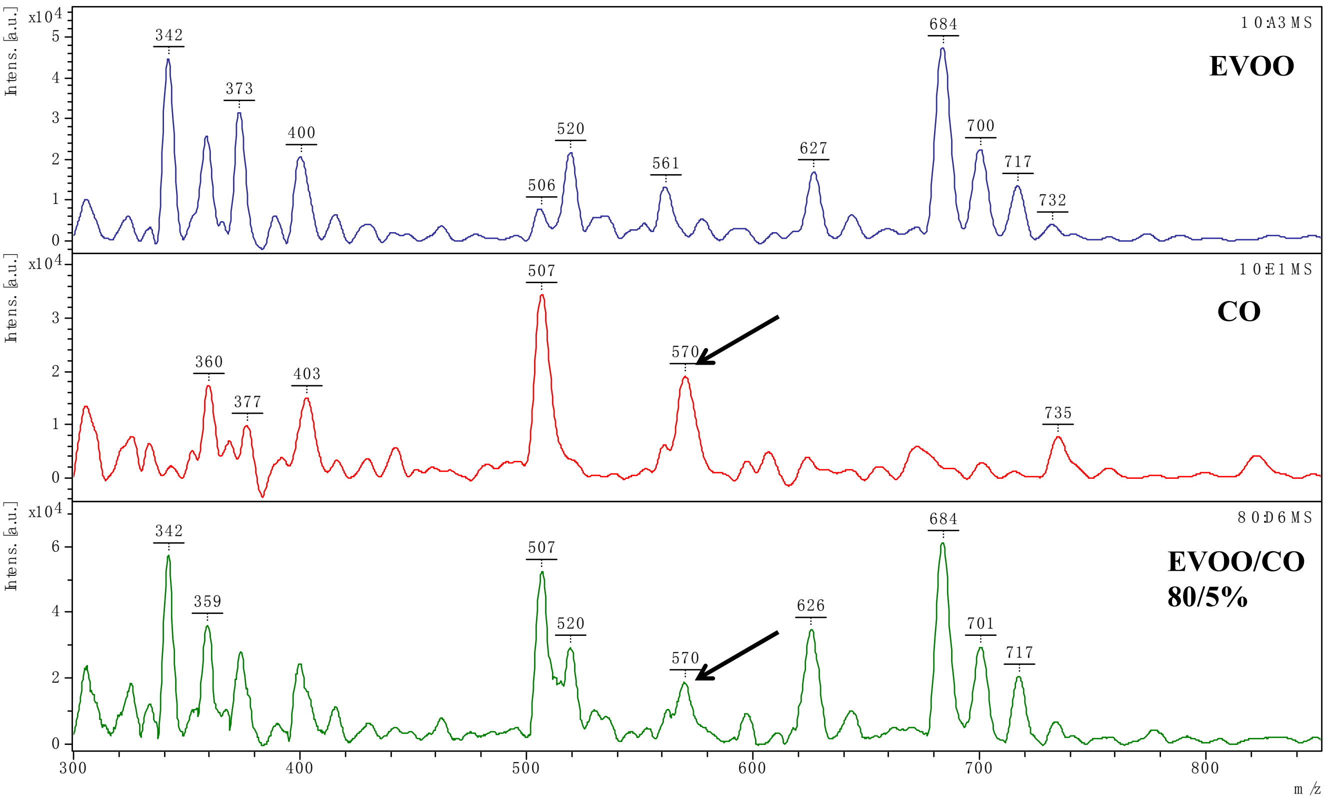

(C)

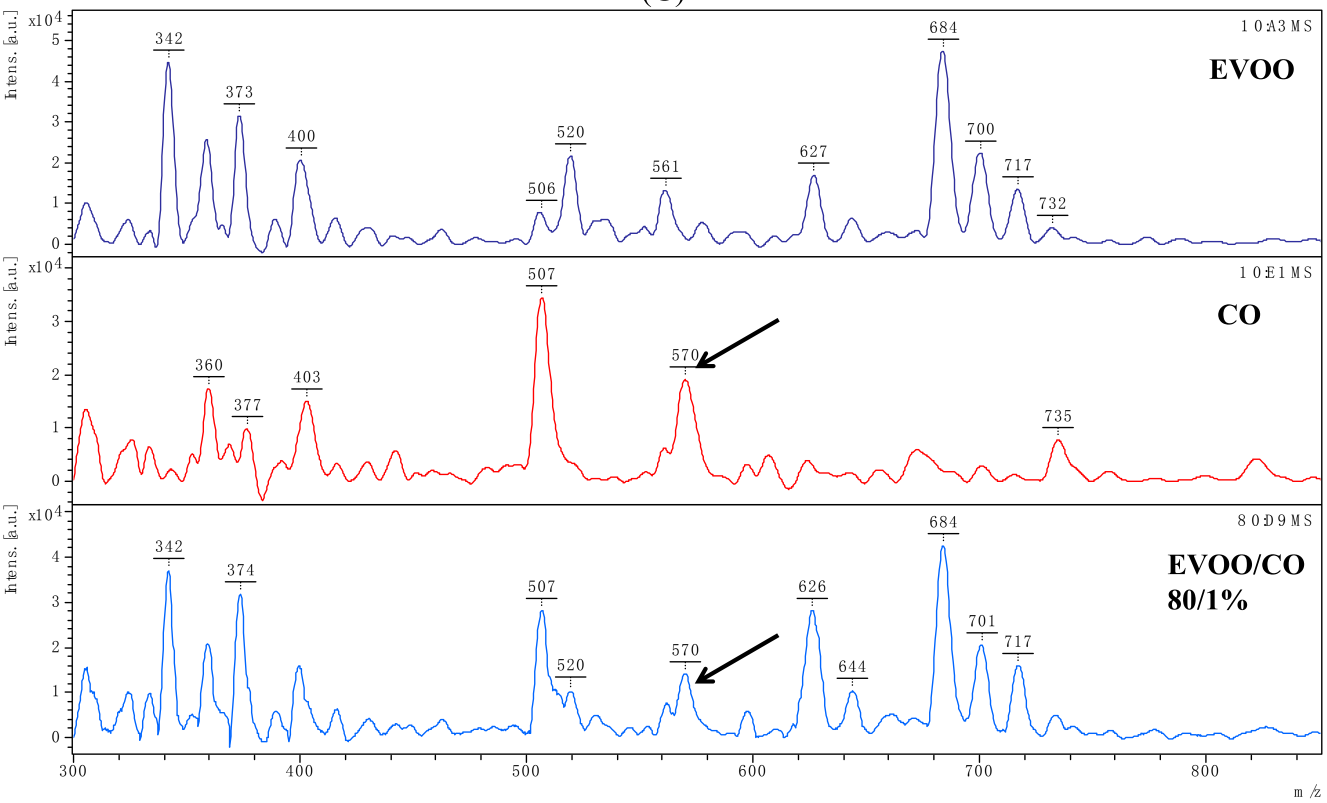

(D)

Figure S2. *Cont.*

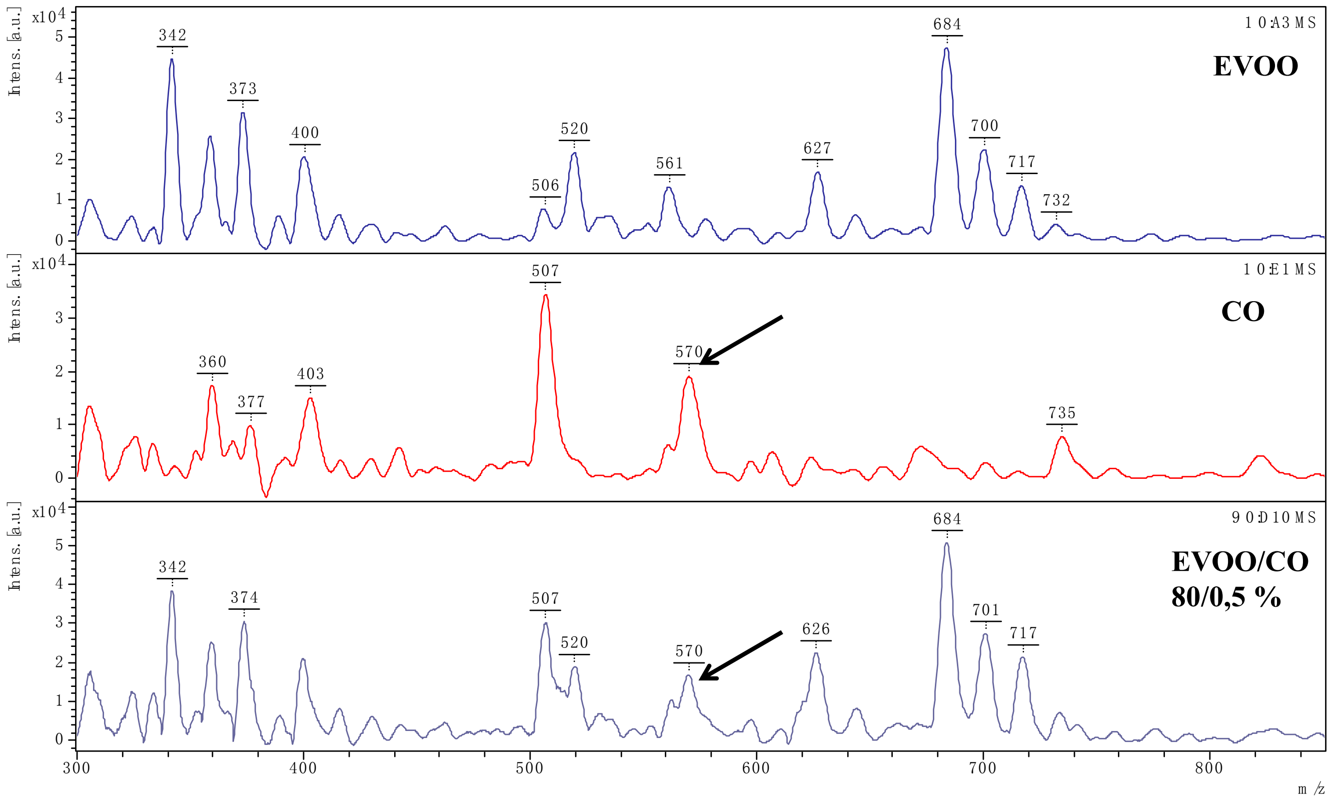

(E)

Figure S2. The *m*/*z* = 570 (indicated from the arrows) represents the discriminating peak of the all adulteration levels (panels **A**–**E**).
